# Detection of Higher Cycle Threshold Values in Culturable SARS-CoV-2 Omicron BA.1 Sublineage Compared with Pre-Omicron Variant Specimens — San Francisco Bay Area, California, July 2021—March 2022

**DOI:** 10.15585/mmwr.mm7136a3

**Published:** 2022-09-09

**Authors:** Michel Tassetto, Miguel Garcia-Knight, Khamal Anglin, Scott Lu, Amethyst Zhang, Mariela Romero, Jesus Pineda-Ramirez, Ruth Diaz Sanchez, Kevin C. Donohue, Karen Pfister, Curtis Chan, Sharon Saydah, Melissa Briggs-Hagen, Michael J. Peluso, Jeffrey N. Martin, Raul Andino, Claire M. Midgley, J. Daniel Kelly

**Affiliations:** ^1^Department of Microbiology and Immunology, University of California at San Francisco, San Francisco, California; ^2^Institute for Global Health Sciences, University of California at San Francisco, San Francisco, California; ^3^School of Medicine, University of California at San Francisco, San Francisco, California; ^4^San Mateo County Health, San Mateo, California; ^5^CDC COVID-19 Emergency Response Team; ^6^Division of HIV, Infectious Diseases, and Global Medicine, Zuckerberg San Francisco General Hospital, University of California at San Francisco, San Francisco, California; ^7^Department of Epidemiology and Biostatistics, University of California at San Francisco, San Francisco, California; ^8^Francis I. Proctor Foundation for Research in Ophthalmology, University of California at San Francisco, San Francisco, California.

Before emergence in late 2021 of the highly transmissible B.1.1.529 (Omicron) variant of SARS-CoV-2, the virus that causes COVID-19 (*1*,*2*), several studies demonstrated that SARS-CoV-2 was unlikely to be cultured from specimens with high cycle threshold (Ct) values^§^ from real-time reverse transcription–polymerase chain reaction (RT-PCR) tests (suggesting low viral RNA levels) (*3*). Although CDC and others do not recommend attempting to correlate Ct values with the amount of infectious virus in the original specimen (*4*,*5*), low Ct values are sometimes used as surrogate markers for infectiousness in clinical, public health, or research settings without access to virus culture (*5*). However, the consistency in reliability of this practice across SARS-CoV-2 variants remains uncertain because Omicron-specific data on infectious virus shedding, including its relationship with RNA levels, are limited. In the current analysis, nasal specimens collected from an ongoing longitudinal cohort^¶^ (*6*,*7*) of nonhospitalized participants with positive SARS-CoV-2 test results living in the San Francisco Bay Area** were used to generate Ct values and assess for the presence of culturable SARS-CoV-2 virus; findings were compared between specimens from participants infected with pre-Omicron variants and those infected with the Omicron BA.1 sublineage. Among specimens with culturable virus detected, Ct values were higher (suggesting lower RNA levels) during Omicron BA.1 infections than during pre-Omicron infections, suggesting variant-specific differences in viral dynamics. Supporting CDC guidance, these data show that Ct values likely do not provide a consistent proxy for infectiousness across SARS-CoV-2 variants.

As part of an ongoing longitudinal cohort study, persons with documented SARS-CoV-2 infection (based on a positive clinical real-time RT-PCR test result) and their household members were recruited within 5 days of the first symptom onset in the household (or first RNA-positive test result if the infected person was asymptomatic). All participants self-collected nasal swab specimens once daily for 2 weeks from the first onset in the household; some participants also provided a serum specimen at enrollment to identify evidence of previous infection.^††^ In a single laboratory, real-time RT-PCR targeting SARS-CoV-2 nucleocapsid (*N*) and envelope protein (*E*) genes^§§^ (*8*) was used to detect RNA and to determine Ct values, whole genome sequencing was used to identify the infecting variant strain and sublineage, and the presence or absence of culturable virus was assessed by cytopathic effect observed in tissue culture.^¶¶^ Enrollment sera were tested for the presence or absence of anti-N immunoglobulin G (IgG) per manufacturer (Abbott) instructions at a clinical laboratory at the University of California, San Francisco.

Participants with confirmed infection (based on having at least one nasal specimen test positive by real-time RT-PCR for both *N* and *E*) were included, and the analysis was limited to specimens collected within 14 days of onset for each participant (for symptomatic patients, onset was defined as the first day of symptoms,*** and for asymptomatic participants, as the first RNA-positive specimen [i.e., positive for both *N* and *E* real-time RT-PCR targets]). Participants aged ≥18 years were classified as adults, and those aged <18 years were classified as children and adolescents. Vaccination status was classified as fully vaccinated^†††^ (completion of a primary COVID-19 vaccination series) or unvaccinated; no participants were partially vaccinated, and no participants had received a booster dose ≥14 days before either symptom onset or enrollment. Ct values of Omicron specimens were compared with those of pre-Omicron specimens among all specimens, among RNA-positive specimens, and among specimens with viable virus detected in tissue culture (virus-positive specimens). With *E*-specific Ct value as the main outcome and variant group (Omicron versus pre-Omicron) as the main exposure, mixed linear regression models were used to account for clustering of multiple specimens per participant, and to control for potential confounding by age group and vaccination status. When Ct values among all or RNA-positive specimens were compared, an interaction term of the product of variant and infectiousness (i.e., virus-positivity) was included; this interaction term was excluded when Ct values within virus-positive specimens were assessed. Longitudinal sampling of infected participants resulted in some subsequently negative real-time RT-PCR specimens (no target detected); these were included in the all-specimen models and were assigned a Ct value of 40 for analysis. Sensitivity analyses were conducted with comparable models using *N*-specific Ct values as the outcome. All statistical analyses were performed using Stata Software (version 16.1; StataCorp). This activity was reviewed by CDC and was conducted consistent with applicable federal law and CDC policy.^§§§^

A total of 1,147 nasal swab specimens from 124 participants were analyzed; among 17 participants infected with Omicron variants (all BA.1 sublineages) and 107 infected with pre-Omicron variants,^¶¶¶^ 149 and 998 specimens, respectively, were collected (Table). Timing of specimen collection after onset (in each participant) was similar in both groups (median = 8 days; IQR = 6–11 days). Among the 17 participants with Omicron BA.1 infections, nine (53%) were adults and 10 (59%) were fully vaccinated. Among 107 participants with pre-Omicron infections, 92 (86%) were adults and 35 (33%) were fully vaccinated. Nearly all participants were symptomatic (16 of 17 participants with Omicron BA.1 infection and 100 of 107 with pre-Omicron infection). No participants reported previous infection, and among 58 participants with available sera, none had detectable anti-N IgG at enrollment.

**TABLE:** Characteristics of participants infected with SARS-CoV-2 pre-Omicron variants and Omicron BA.1 sublineage and nasal swab specimens evaluated for real-time reverse transcription–polymerase chain reaction cycle threshold values — San Francisco Bay Area, California, July 2021–March 2022

Participant and specimen	No. (%)		Change in *E*-specific Ct value between pre-Omicron and Omicron variants
	Pre-Omicron	Omicron	
**All participants (N = 124)**	**107 (100)**	**17 (100)**	—
Adults aged ≥18 yrs	92 (86)	9 (53)	—
Fully vaccinated*	35 (33)	10 (59)	—
Symptomatic^†^	100 (93)	16 (94)	—
Culturable virus detected	76 (71)	13 (76)	—
**All specimens (N = 1,147)**	**998 (100)**	**149 (100)**	**4.45^§^**
RNA-positive specimens^¶^	539 (53)	72 (48)	3.90^§^
Virus-positive specimens^¶^	298 (30)	39 (26)	5.77^§^
Median duration of virus detection after onset, days (IQR)	6 (5–8)	6 (5–8)	—
Median interval from onset to specimen collection, days (IQR)	8 (6–11)	8 (6–11)	

**Abbreviations:** Ct = cycle threshold; *E* = envelope gene.

* Fully vaccinated participants were defined as those who had received all recommended doses of a Food and Drug Administration–authorized or approved primary vaccine series (2 mRNA vaccine doses or a single dose of Johnson & Johnson [Janssen] vaccine) ≥14 days before either symptom onset or enrollment (whichever occurred earlier).

^†^ Participants were considered symptomatic if they reported one or more COVID-19 signs or symptoms consistent with those listed by CDC, including fever, chills, shortness of breath, fatigue, muscle or body aches, headache, loss of taste, loss of smell, sore throat, congestion, runny nose, nausea, vomiting, or diarrhea. https://www.cdc.gov/coronavirus/2019-ncov/symptoms-testing/symptoms.html

^§^ p<0.001.

^¶^ RNA-positive specimens are positive for both SARS-CoV-2 nucleocapsid and envelope gene real-time RT-PCR targets. Virus-positive specimens contain viable SARS-CoV-2 virus detected in tissue culture.

Accounting for age group and vaccination status, *E*-specific Ct values in all specimens were significantly higher in Omicron specimens than in pre-Omicron specimens (Ct difference = 4.45, p<0.001).**** When analysis was limited to RNA-positive specimens, a similar trend was observed (Ct difference = 3.90, p<0.001).^††††^ Despite these higher Ct values in Omicron than in pre-Omicron specimens, culturable virus was detected in specimens from a similar percentage of participants in both variant groups (Omicron = 76%; pre-Omicron = 71%), a similar percentage of total specimens (Omicron = 26%; pre-Omicron: 30%), and was detected for a similar duration following onset (median = 6 days, IQR = 5–8 days for both Omicron and pre-Omicron specimens). Among virus-positive specimens, *E*-specific Ct values were significantly higher in Omicron specimens than pre-Omicron specimens (Ct difference = 5.77, p<0.001).^§§§§^ This difference was observed as early as day 3 after onset through day 8 after onset (Figure 1). When stratified by age group or vaccination status (Figure 2), virus-positive Omicron specimens were associated with higher *E*-specific Ct values than were virus-positive pre-Omicron specimens (p<0.01). Similar findings were observed in the *N*-specific analysis (p<0.001).

**FIGURE 1:**
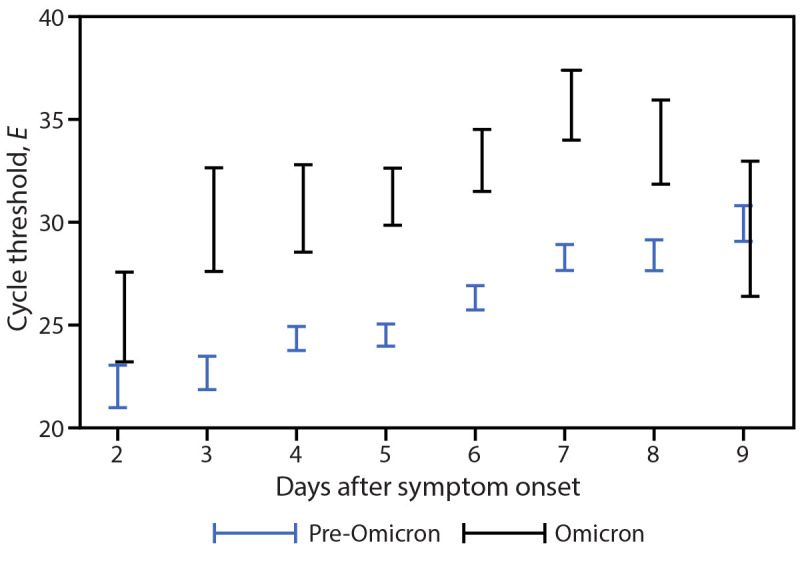
Pre-Omicron and Omicron BA.1 envelope gene–specific* cycle threshold values among nasal specimens with culturable SARS-CoV-2 virus,^†^^,^^§^ by days after illness onset — San Francisco Bay Area, California, July 2021–March 2022 **Abbreviation:** *E* = envelope gene. * Nucleocapsid-specific real-time reverse transcription–polymerase chain reaction results were similar. ^†^ Included 33 Omicron specimens and 256 pre-Omicron specimens. ^§^ Displayed as 95% CIs. The mixed model used in this analysis included an interaction term between variant and time after symptom onset.

**FIGURE 2:**
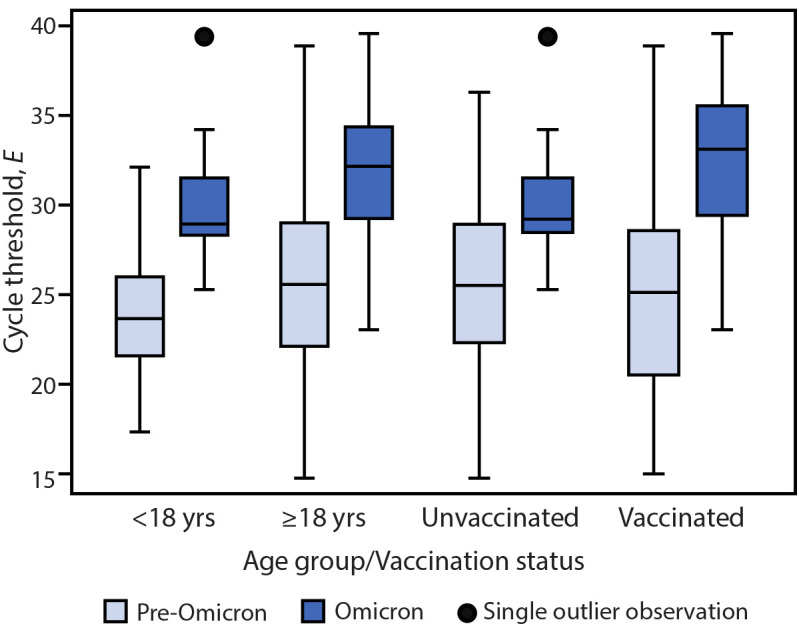
Pre-Omicron and Omicron BA.1 envelope gene–specific* cycle threshold values among nasal specimens with culturable SARS-CoV-2 virus,^†^ by age group^§^ and by primary COVID-19 vaccination status^¶^^,^** — San Francisco Bay Area, California, July 2021–March 2022 **Abbreviation:** *E* = envelope gene. * Nucleocapsid-specific real-time reverse transcription–polymerase chain reaction results were similar. ^†^ Displayed as 95% CIs. The mixed model used in this analysis included an interaction term between variant and time after symptom onset. ^§^ Adults aged ≥18 years included 21 Omicron and 273 pre-Omicron specimens. Children and adolescents aged <18 years included 18 Omicron and 25 pre-Omicron specimens. ^¶^ Fully vaccinated included 18 Omicron and 81 pre-Omicron specimens. Unvaccinated included 21 Omicron and 217 pre-Omicron specimens. ** Boxplots display the median, lower, and upper quartiles and 1.5 times above or below the lower and upper quartiles.

## Discussion

In this study, and consistent with other published findings (*9*), Ct values detected in nasal specimens were higher (suggesting lower RNA levels) in those obtained from participants infected with SARS-CoV-2 Omicron BA.1 sublineage than in those from participants infected with pre-Omicron variants. However, despite these higher Ct values, culturable virus was detected from a similar proportion of participants in both variant groups, and for a similar duration following onset; consistent with a recent report (*10*), participants infected with Omicron BA.1 had detectable culturable virus for a median of 6 days after onset. Notably, among these virus-positive (i.e., potentially infectious) specimens, Ct values were higher than were those for pre-Omicron specimens, especially during the first week of illness. In addition, these differences between Omicron and pre-Omicron infections were observed in adults and in children and adolescents and were irrespective of vaccination status. Presence of culturable Omicron BA.1 in nasal specimens, despite high Ct values, might contribute to the high levels of Omicron transmission observed in other studies (*2*). Further, these findings highlight variant-specific differences in viral dynamics, specifically, differences in the relationship between RNA and shedding of infectious virus. 

Strengths of this study include the robust prospective longitudinal nature of nasal swab specimen collection. Similar findings were observed from two distinct real-time RT-PCR targets, both of which have been shown to reliably amplify both Omicron and pre-Omicron variants. 

The findings in this report are subject to at least three limitations. First, this is a single-site study with a small number of participants infected with the Omicron BA.1 sublineage; thus, these findings might not be representative of all infected persons. Replication of these findings with additional participants is necessary and is ongoing. Second, approximately one half of the participants did not provide an enrollment serum specimen; thus, it was not possible to comprehensively assess the incidence of previous infection. Finally, duplication was not carried out on multiple real-time RT-PCR platforms across laboratories.

Virus-positive (i.e., potentially infectious) specimens from participants infected with SARS-CoV-2 Omicron variants had significantly higher Ct values than did virus-positive specimens from participants infected with pre-Omicron variants. Supporting CDC guidance (*4*), these data highlight that Ct values likely do not provide a reliable or consistent proxy for infectiousness across SARS-CoV-2 variants.

**Summary**

**What is already known about this topic?**

Before emergence of the SARS-CoV-2 B.1.1.529 (Omicron) variant, infectious SARS-CoV-2 was unlikely to be cultured at high cycle threshold (Ct) values. Based on this, low Ct values, which are suggestive of high RNA levels, are sometimes used as surrogate markers for infectiousness.

**What is added by this report?**

In a longitudinal study including daily nasal swabbing, although Omicron BA.1 sublineage infections exhibited higher Ct values than did pre-Omicron infections, culturable Omicron virus was still detected. Among virus-positive specimens, Ct values were higher for Omicron than for pre-Omicron specimens, especially during the first week of illness.

**What are the implications for public health practice?**

Supporting CDC guidance, these data show that Ct values likely do not provide a consistent proxy for infectiousness across SARS-CoV-2 variants.

## Footnotes

* These authors contributed equally to this report.

† These senior authors contributed equally to this report.

§ Ct values reflect the number of amplification cycles necessary to detect viral RNA. Ct values are inversely related to the amount of viral RNA present in a specimen and are sometimes used as a proxy for viral RNA levels or loads, with low Ct values indicating high viral RNA levels and high Ct values low viral RNA levels. Ct value of 40 was assigned to negative real-time RT-PCR results because the PCR was run for 40 cycles without detecting any signal.

¶ In brief, study enrollment began during September 2020. Eligible persons with positive SARS-CoV-2 RT-PCR test results are identified from local outpatient or public health COVID-19 data sources and contacted to ask about interest in the study. Persons are eligible if they can be enrolled within 5 days of their illness onset (or first positive test result, if asymptomatic), and have at least one household member who is also willing to enroll and is not yet known to be infected themselves; if a household member is already known to be infected, that household can still be eligible if household members can be enrolled within 5 days of that household member’s illness onset (or first positive test result, if asymptomatic). At enrollment, participants provide an optional blood specimen that is taken to a biorepository that same day for processing as serum, aliquoting, and long-term storage at −112°F (−80°C).

** The San Francisco Bay Area consists of nine counties (Alameda, Contra Costa, Marin, Napa, San Francisco, San Mateo, Santa Clara, Solano, and Sonoma) and 101 municipalities in California.

†† Participants are instructed on how to collect (daily) and store nasal specimens; daily specimens are collected regardless of symptom status. Once collected, nasal specimens are stored at −4°F (−20°C) in the participant’s household until transferred on dry ice to a biorepository for processing, aliquoting, and long-term storage at −112°F (−80°C).

§§ Detection of these targets is not affected by the viral genome sequences of the variants, such as Omicron. The *E* gene is not mutated between Omicron and pre-Omicron genomes, and the only mutation in the targeted sequence of the real-time RT-PCR probe of the Omicron *N* gene does not perturb its efficient detection.

¶¶ Cytopathic effect (CPE) was assessed in Vero cells stably overexpressing the human TMPRSS-2 and ACE-2 genes. Briefly, 200 *μ*L of nasal specimen (3 mL total specimen, previously aliquoted and frozen at −112°F [−80°C]) were added to a well of a 96-well plate and serially diluted twofold with 2.5x104 cells per well. Vero-TMPRSS2-hACE2 cells form characteristic syncytia (fused cells) upon SARS-CoV-2 infection, enabling rapid and specific visual evaluation for CPE, which was assessed after 2 and 5 days. Cells from wells with CPE were then processed for RNA extraction and real-time RT-PCR to confirm SARS-CoV-2 infection.

*** Participants were considered symptomatic if they reported one or more COVID-19 signs or symptoms consistent with those listed by CDC, including fever, chills, shortness of breath, fatigue, muscle or body aches, headache, loss of taste, loss of smell, sore throat, congestion, runny nose, nausea, vomiting, or diarrhea. https://www.cdc.gov/coronavirus/2019-ncov/symptoms-testing/symptoms.html

††† Fully vaccinated participants were defined as those who had received all recommended doses of a Food and Drug Administration–authorized or approved primary vaccine series (2 mRNA vaccine doses or a single dose of Johnson & Johnson [Janssen] vaccine) ≥14 days before either symptom onset or enrollment (whichever occurred earlier).

§§§ 45 C.F.R. part 46.102(l)(2), 21 C.F.R. part 56; 42 U.S.C. Sect. 241(d); 5 U.S.C. Sect. 552a; 44 U.S.C. Sect. 3501 et seq.

¶¶¶ Pre-Omicron infections included 40 B.1.617.2 (Delta) and 67 pre-Delta infections.

**** Among all specimens, *N*-specific Ct values were also significantly higher in Omicron versus pre-Omicron infections (Ct difference = 3.84, p<0.001).

†††† Among RNA-positive specimens, *N*-specific Ct values were also significantly higher in Omicron versus pre-Omicron infections (Ct difference = 3.27, p<0.001).

§§§§ Among virus-positive specimens, *N*-specific Ct values were also significantly higher in Omicron versus pre-Omicron infections (Ct difference = 3.84, p<0.001). Further, no difference was detected in either *E-* or *N*-specific Ct values over time between Delta and pre-Delta specimens (*E*: Ct difference = −1.86, p = 0.25; *N*: Ct difference = −2.35, p = 0.14). Although not statistically significant, in the case of Delta infections, *E*- and *N*-specific Ct values were lower (suggesting higher viral RNA levels) compared with pre-Delta infections, hence the negative difference between Delta and pre-Delta specimens.
